# A case of migraine misdiagnosed as epilepsy

**DOI:** 10.1186/s42494-022-00112-1

**Published:** 2023-02-01

**Authors:** Yuting Yang, Xi Peng, Yangmei Chen

**Affiliations:** Chongqing Medical University, Chongqing, 400010 China

**Keywords:** Epilepsy, Migraine, Electroencephalogram

## Abstract

**Background:**

Migraine and epilepsy are both episodic disorders, with some overlapping symptoms, mechanisms and therapies. Clinically, there is a comorbid relationship between them. Some migraine patients may exhibit epileptic discharges by electroencephalogram (EEG) recording. Therefore, the two conditions are easily misdiagnosed.

**Case presentation:**

We reported a 16-year-old female patient who was admitted to our hospital due to repeated headaches with disturbance of consciousness. Epileptic discharge was recorded by long-term EEG. The patient had been misdiagnosed as epilepsy, and had no response to anti-seizure medicines (ASMs). We revised her diagnosis and prescribed ibuprofen in her acute episode and prophylactic medicine, including flunarizine and amitriptyline in her interictal period. One week later, her headache disappeared.

**Conclusions:**

This patient manifested with altered levels of consciousness during headache episodes, and the abnormal EEG results lead to the misdiagnosis as epilepsy. Clinicians should be cautious to the distinction between migraine and epilepsy.

## Background

Migraine and epilepsy are both episodic disorders and share some clinical features. Epidemiologic studies have shown a comorbid relationship between the two conditions [1]. There is a higher incidence of migraine in epileptic patients compared with the general population, and vice versa. Migraine and epilepsy are considered as disorders of neuronal hyperexcitability, and the most common pathological mechanism is ion channel dysfunction. Some migraine patients may suffer epileptic discharges as recorded by electroencephalogram (EEG) [2]. Due to the overlapping disease features, migraine and epilepsy may be mistaken one for the other in clinical works. Here we report a patient with migraine who was misdiagnosed as epilepsy and failed to respond to anti-seizure medicine (ASM) treatment.

## Case presentation

**Fig. 1:**
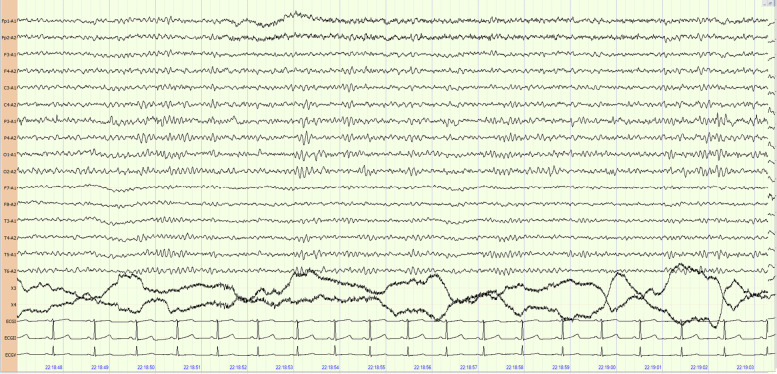
Electroencephalographic background the electroencephalogram was recorded in a quiet state when the patient was awake, with eyes closed, on the background of 9–10 Hz and 10–50 μV α wave. The EEG background was normal

**Fig. 2:**
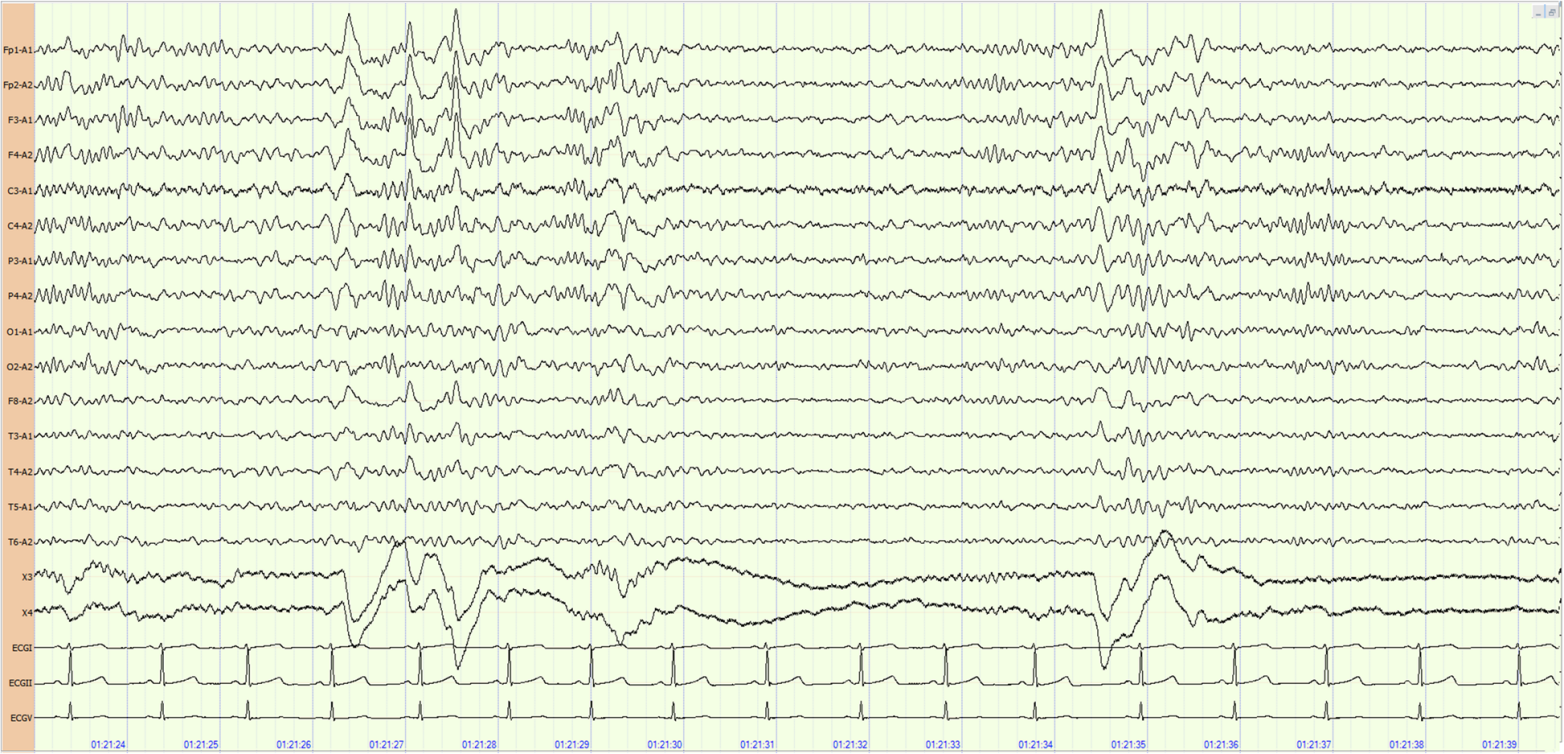
EEG recording in the non-rapid eye movement (NREM) I stage. High amplitude slow waves and spikes over the former head (focused on frontal area)

A 16-year-old female patient was admitted to our hospital for repeated headache with disturbance of consciousness for 3 years. Three years ago, she suffered from a sudden headache, which was described as severe throbbing pain located in her bilateral temporal and occipitoparietal areas, accompanied by vertigo. After several minutes, she could not speak and fell down, with her eyes closed and face flushed. The symptom lasted for 15 min, and then she woke up by herself. After waking up, she felt persistent throbbing pain, nausea, photophobia, and palpitation. These symptoms disappeared 6 h later and she recovered. During the past 3 years, the symptoms mentioned above relapsed frequently, from 2 to 5 times per month. The symptoms lasted for a number of hours or a day each time. During the ictal period, she could hear the sound but could not speak completely. Her eyes were staring off, and she displayed frothing at the mouth, tongue bite, orthocolosis or gatism. All episodes were present in the daytime. Long-term EEG monitoring in the interictal period indicates frequent discharges of moderate-to-high-amplitude sharp slow waves in the bilateral frontal and temporal lobes during waking and sleeping times. According to the symptoms and EEG findings, the patient was diagnosed with partial epilepsy, and was treated with levetiracetam at the dose of 500 mg twice a day. However, she had no response to levetiracetam. After one year treatment of levetiracetam, she was transferred to another hospital and given oxcarbazepine at 300 mg twice a day as an add-on therapy. After taking two ASMs for 1 year, she was getting worse. The frequency of episode increased to four times a week. Then she stopped the ASMs, and the severity and frequency of episode did not change significantly. After that, she came to our hospital for further treatment. She had an average level of intelligence, a cheerful personality, and no bad habits; She did not have severe illnesses, nor did she receive operations in the past. Her mother had headache for many years without treatments. Physical examinations did not show any abnormality. Blood cell counts, erythrocyte sedimentation rates, electrolytes and blood biochemistry were normal. The brain magnetic resonance imaging (MRI), computed tomography angiography, CT venography, and Holter monitoring had no abnormal findings. The video EEG recording did not capture the ictal period. Her interictal EEG showed normal background (Fig. 1). But in the early drowsiness period, there is intermittent moderate-to-high-amplitude sharp slow waves in her bilateral frontal and temporal lobes (Fig. 2). Although the patient had an abnormal interictal EEG, the diagnosis of epilepsy was suspicious. The patient had headache with brain stem aura symptoms (vertigo, etc.) before fainting, with no increased muscle tension or convulsions during fainting. The headache during the whole process was a significant symptom, and the nature of the headache was consistent with the diagnosis of migraine. In addition, the patient did not respond to antiepileptic drugs. Therefore, she was diagnosed with migraine with brainstem aura and was given 300 mg ibuprofen in her acute episode, 10 mg flunarizine at night and 12.5 mg amitriptyline twice a day to prevent migraine. After one week, her symptoms disappeared. The flunarizine and amitriptyline treatment was continued for 3 months. She got episode free for more than 1 year.

## Discussion

Migraines are a broad spectrum of brain disorders classified by the type of aura. The migraine with brainstem aura is a rare subtype, which was previously described as basilar artery migraine, basilar migraine, and basilar-type migraine, and was renamed as migraine with brainstem aura in International Classification of Headache Disorders, 3rd edition (ICHD-3) (Headache Classification Committee of the International Headache Society, 2013) and maintained in the final ICHD-3 in 2018. To fulfill the diagnostic criteria for migraine with brainstem aura according to the ICHD-3, an individual should suffer attacks of migraine with aura and at least two brainstem aura symptoms consisting of dysarthria, vertigo, tinnitus, hypacusis, diplopia, ataxia and decreased level of consciousness. In our case, the migraine with brainstem aura was manifested as a few minutes of vertigo and disturbance of consciousness during headaches. These symptoms of headache matched the characteristics of migraine.

Why was this patient misdiagnosed as epilepsy? The main reason was her abnormal EEG. Although EEG has a well-established role in the diagnosis of epilepsy, not all abnormal EEGs represent epilepsy [3, 4]. The abnormal EEG also can be present in patients with migraine [1]**.** Some researchers retrospective analyzed 259 patients with migraine and found that 31 showed abnormal EEG and the frequency of EEG abnormalities was significantly high in patients having migraines with auras than those without auras [5]. The abnormal EEGs include epileptiform discharges (focal and generalized spikes) and slowing waves. Bursts of slower brain electrical activity have been shown during and after migrainous headaches, and scattered sharp activity has also been recorded, and scattered sharp activity has also been noted**.** A strong high-frequency photostimulation can be associated with migraine [6, 7].

Migraine and epilepsy are both common paroxysmal and chronic brain disorders, with several overlapping clinical features including triggers, inherited tendency, transient neurological symptoms, and autonomic and psychological symptoms, and they both had normal interattacks [8]. During a migraine attack, patients may experience mental confusion or disturbance of consciousness, while epileptic patients may suffer preictal, ictal or postictal headache. Migraine aura can also induce seizure [9]. As to the disease incidence, the incidence of migraine is 5–10% in the general population, and rises up to 8–23% in patients with epilepsy. Similarly, the incidence of epilepsy in the general population is between 0.5–1%, but in migraine patients it is 5.9% [8]

The differential diagnosis of migraine and epilepsy is another keypoint. Migraine visual aura may present as a amorphous flash or dark spot in the visual field, whereas the epileptic visual auras are characterized by colored circular patterns or complex and vivid scenes [10]. migraine auras last for several minutes, while the duration of epileptic auras are shorter, mostly less than a minute. According to the ICHD3-beta criteria, a migraine aura is defined to have visual symptoms lasting longer than 5 minutes [11]. Sensory auras exist in both migraine and epilepsy; The migration of prickling sensation corresponds to the sensory cortex, but lasts much longer in migraine [12]. The same goes for temporal lobe epilepsy, which is accompanied by auras of gustatory and olfactory sensations, or gastric symptoms, that only present for a few seconds, followed by complex partial seizures, whereas these migraine-related sensation auras may last for hours to days [13]. However, the duration of symptoms is not always helpful in distinguishing between the two diseases. Both hemiplegic migraine and Todd’s paralysis can last from hours to days, and the symptom of dysphasic can vary widely in duration.

## Conclusions

Migraine and epilepsy have overlapping symptoms, including abnormal EEG. Therefore, migraine may be misdiagnosed as epilepsy. Migraine with brainstem aura may have brainstem-related symptoms, which should be distinguished from focal epileptic seizures. Our patient experienced altered levels of consciousness during headache episodes, and the abnormal EEG results led to misdiagnosis as epilepsy. Therefore, clinicians should pay attention to distinguishing between migraine and epilepsy.

## Data Availability

Not applicable.

## Abbreviations

ASMs: Anti-seizure medicines
EEG: Electroencephalogram
ICHD-3: International Classification of Headache Disorders, 3rd edition
NREM: Non-rapid eye movement
